# A pooled analysis of magnetic fields and childhood leukaemia

**DOI:** 10.1054/bjoc.2000.1376

**Published:** 2000-08-16

**Authors:** A Ahlbom, N Day, M Feychting, E Roman, J Skinner, J Dockerty, M Linet, M McBride, J Michaelis, J H Olsen, T Tynes, P K Verkasalo

**Affiliations:** Division of Epidemiology, National Institute of Environmental Medicine, Karolinska Institute, Sweden; Strangeways Research Laboratory, University of Cambridge, UK; Leukaemia Research Fund Centre for Clinical Epidemiology, University of Leeds, UK; Childhood Cancer Research Group, University of Oxford, UK; Division of Cancer Epidemiology and Genetics, National Cancer Institute, USA; Cancer Control Research Programme, British Columbia Cancer Agency, Canada; Institute of Medical Statistics and Documentation, University of Mainz, Grmany; Institute of Cancer Epidemiology, Danish Cancer Society, Denmark; Institute of Epidemiological Cancer Research, Norway; Department of Public Health, University of Helsinki, Finland; Finnish Cancer Registry; Department of Public Health, University of Turku, Finland

**Keywords:** EMF, cancer, childhood leukaemia, meta-analysis, pooled analysis, epidemiology

## Abstract

Previous studies have suggested an association between exposure to 50–60 Hz magnetic fields (EMF) and childhood leukaemia. We conducted a pooled analysis based on individual records from nine studies, including the most recent ones. Studies with 24/48-hour magnetic field measurements or calculated magnetic fields were included. We specified which data analyses we planned to do and how to do them before we commenced the work. The use of individual records allowed us to use the same exposure definitions, and the large numbers of subjects enabled more precise estimation of risks at high exposure levels. For the 3203 children with leukaemia and 10 338 control children with estimated residential magnetic field exposures levels < 0.4 μT, we observed risk estimates near the no effect level, while for the 44 children with leukaemia and 62 control children with estimated residential magnetic field exposures ≥ 0.4 μT the estimated summary relative risk was 2.00 (1.27–3.13), *P* value = 0.002). Adjustment for potential confounding variables did not appreciably change the results. For North American subjects whose residences were in the highest wire code category, the estimated summary relative risk was 1.24 (0.82–1.87). Thus, we found no evidence in the combined data for the existence of the so-called wire-code paradox. In summary, the 99.2% of children residing in homes with exposure levels < 0.4 μT had estimates compatible with no increased risk, while the 0.8% of children with exposures ≥ 0.4 μT had a relative risk estimate of approximately 2, which is unlikely to be due to random variability. The explanation for the elevated risk is unknown, but selection bias may have accounted for some of the increase. © 2000 Cancer Research Campaign


*British Journal of Cancer* (2000) **83**(5), 692–698
© 2000 Cancer Research Campaign
doi: 10.1054/ bjoc.2000.1376, available online at http://www.idealibrary.com on IDEAL®

### A pooled analysis of magnetic fields and childhood leukaemia

**A Ahlbom[1], N Day[2], M Feychting[1], E Roman[3], J Skinner[2], J Dockerty[4], M Linet[5], M McBride[6], J Michaelis[7], JH Olsen[8], T Tynes[9] and PK Verkasalo[10,11,12]**

[1]Division of Epidemiology, National Institute of Environmental Medicine, Karolinska Institute, Sweden; [2]Strangeways Research Laboratory, University of Cambridge, UK; [3]Leukaemia Research Fund Centre for Clinical Epidemiology, University of Leeds, UK; [4]Childhood Cancer Research Group, University of Oxford, UK; [5]Division of Cancer Epidemiology and Genetics, National Cancer Institute, USA; [6]Cancer Control Research Programme, British Columbia Cancer Agency, Canada; [7]Institute of Medical Statistics and Documentation, University of Mainz, Germany; [8]Institute of Cancer Epidemiology, Danish Cancer Society, Denmark; [9]Institute of Epidemiological Cancer Research, Norway; [10]Department of Public Health, University of Helsinki, Finland; [11]Finnish Cancer Registry; [12]Department of Public Health, University of Turku, Finland

**Summary** Previous studies have suggested an association between exposure to 50–60 Hz magnetic fields (EMF) and childhood leukaemia. We conducted a pooled analysis based on individual records from nine studies, including the most recent ones. Studies with 24/48-hour magnetic field measurements or calculated magnetic fields were included. We specified which data analyses we planned to do and how to do them before we commenced the work. The use of individual records allowed us to use the same exposure definitions, and the large numbers of subjects enabled more precise estimation of risks at high exposure levels. For the 3203 children with leukaemia and 10 338 control children with estimated residential magnetic field exposures levels < 0.4 μT, we observed risk estimates near the no effect level, while for the 44 children with leukaemia and 62 control children with estimated residential magnetic field exposures ≥ 0.4 μT the estimated summary relative risk was 2.00 (1.27–3.13), *P* value = 0.002). Adjustment for potential confounding variables did not appreciably change the results. For North American subjects whose residences were in the highest wire code category, the estimated summary relative risk was 1.24 (0.82–1.87). Thus, we found no evidence in the combined data for the existence of the so-called wire-code paradox. In summary, the 99.2% of children residing in homes with exposure levels < 0.4 μT had estimates compatible with no increased risk, while the 0.8% of children with exposures ≥ 0.4 μT had a relative risk estimate of approximately 2, which is unlikely to be due to random variability. The explanation for the elevated risk is unknown, but selection bias may have accounted for some of the increase. © 2000 Cancer Research Campaign

**Keywords**: EMF; cancer; childhood leukaemia; meta-analysis; pooled analysis; epidemiology

It is now twenty years since Wertheimer and Leeper (1979) published the first study suggesting an association between residential exposure to extremely low frequency magnetic fields (EMF) and childhood cancer. Ever since, this has been a controversial issue with the findings from several, but not all, subsequent epidemiological studies being consistent with an association, particularly with respect to residential exposure and childhood leukaemia (Portier and Wolfe, 1998). However, many of the reports have been based on small numbers of exposed cases, and despite intense experimental research no known biophysical mechanism to explain an effect has been established.

We conducted a pooled analysis based on primary data from nine studies on EMF and childhood leukaemia, addressing three specific questions:

1. Do the combined results of these studies indicate that there is an association between EMF exposure and childhood leukaemia risk, which is larger than one would expect from random variability?
2. Does adjustment for confounding from socioeconomic class, mobility, level of urbanization, detached/not detached dwelling, and level of traffic exhaust change the results?
3. Do the combined data support the existence of the so-called wire code paradox, that is, a stronger association between proxy measures of EMF and cancer than between direct measurements and cancer?

### METHODS

The original plan for this project was to include all European studies that addressed the question of an association between EMF and childhood leukaemia and were based on either 24 or 48 hour magnetic field measurements or calculated fields. At the time five such studies were reported (Feychting and Ahlbom, 1993; Olsen et al, 1993; Verkasalo et al, 1993; Tynes and Haldorsen, 1997; Michaelis et al, 1998). In addition, a nationwide childhood cancer study was in progress and near completion in the UK (UKCCS, 1999). Since we were not aware of any other European study to be published in the near future, the inclusion of the UK study would give us a complete set of European studies. We felt that if we could also incorporate new studies from non-European countries this pooled analysis would be up to date and presumably stay current for several years. We were aware of three more studies in other parts of the world with compatible information that were all nearly

*Received 23 May 2000*
*Revised 16 June 2000*
*Accepted 16 June 2000*

*Correspondence to:* A Ahlbom

**Table 1** Relevant characteristics for studies included in the pooled analysis

| | Subjects | | | Exposure measures | | | Matching variables | | | Potential confounders: Common | | Study specific (no. of groups): Measure of social status | | | | | |
|---|---|---|---|---|---|---|---|---|---|---|---|---|---|---|---|---|---|
| | Cases | Controls | Year of diagnosis | Long measurements | Calculated fields | Wire codes | Sex | Year of birth | Area of diagnosis | Detached or other house | Mobility quintile | Social group | Mother's education | Income | Urbanisation | Car exhaust | Other |
| Canada | 272 | 304 | 1990–94 | ✓ | | ✓ | ✓ | ✓ | ✓ | ✓ | ✓ | | 3 | | 2 | | |
| Denmark | 833 | 4746 | 1968–86 | | ✓ | | ✓ | ✓ | | | ✓ | 5 | | | 4 | | |
| Finland[a] | 29 | 1027 | 1974–93 | | ✓ | | ✓ | ✓ | | | | | | | 2 | | |
| Germany | 175 | 409 | 1992–95 | ✓ | | | ✓ | ✓ | ✓ | ✓ | ✓ | 2 | | | 3 | 2 | 2[c] |
| New Zealand | 86 | 80 | 1990–93 | ✓ | | | ✓ | ✓ | | | ✓ | | 5 | | 2 | | |
| Norway | 148 | 572 | 1965–89 | | ✓ | | ✓ | ✓ | ✓ | ✓ | ✓ | 6 | | | 2 | | |
| Sweden | 36 | 508 | 1960–85 | | ✓ | | ✓ | ✓ | ✓ | ✓ | ✓ | 4 | | | 2 | 3 | |
| USA[b] | 595 | 530 | 1989–94 | ✓ | | ✓ | ✓ | ✓ | ✓ | ✓ | ✓ | | | 6 | 4 | | |
| UK | 1073 | 2224 | 1992–96 | ✓ | | | ✓ | ✓ | ✓ | | | 7 | | | | | |

**Specification of exposure information selected for the pooled analysis**

| | |
|---|---|
| Canada | Latest home inhabited before diagnosis for which a 24-hour bedroom measurement was available (may not be same home for long measurement & wire code) |
| Denmark | Latest home inhabited before diagnosis for which a calculated field was available |
| Finland | Calculated field for 12 months prior to diagnosis was provided especially for this exercise (may be average of values for more than one home) |
| Germany | Latest home inhabited before diagnosis (was home at diagnosis for almost all individuals) |
| New Zealand | Home inhabited at diagnosis |
| Norway | Latest home inhabited before diagnosis in which child lived in the power line corridor, field calculated for entire period |
| Sweden | Latest home inhabited before diagnosis in which child lived in the power line corridor, field calculated for entire period |
| USA | Latest home inhabited before diagnosis for which a record was available (may not be same home for long measurement & wire code) |
| UK | Home inhabited at diagnosis (UKCCS selection meant that the child must have lived there for previous 12 months) |

[a]Case control data generated from the original cohort; [b]acute lymphoblastic leukaemia only; [c]East/West Germany.

completed or recently completed, so we could include those too (Linet et al, 1997; Dockerty et al, 1998, 1999; McBride et al, 1999). Table 1 lists the studies and their relevant characteristics. A fourth study was also near completion in Ontario, Canada, but it was decided that since this study did not provide 24-hour indoor measurements, or anything similar to it, the exposure information in this study was not similar enough to justify inclusion (Green et al, 1999a,b). In effect, all large-scale published studies with extended indoor measurements or calculated fields were included in the pooled analysis with the exception of a few studies that were not population based.

The primary analyses reported here were all discussed and agreed upon prior to the commencement of the work. This included diagnostic categories, exposure definitions, time period for evaluation, cut points, confounders, and statistical methods. In addition certain analyses were done to confirm that the findings from these primary analyses were not dependent on these specifications and yet other analyses were done with an exploratory purpose.

This pooled analysis focused on childhood leukaemia, even though several of the studies also included other cancer diagnoses. The US study included only acute lymphocytic leukaemia (ALL). We did analyses both for total leukaemia and for ALL, but for brevity the more detailed results are given for total leukaemia. There was some variation with respect to age groups in the studies, and we decided to use the age interval 0–14 years.

Since we wanted the data to be as consistent as possible across studies, the data that we used from a particular study were sometimes different from those that formed the basis for the original publication from that study. This was particularly the case with the exposure variables (Table 1). In effect, the study-specific results that we report in this article differ to various degrees from the results as reported in the original publications. These differences are biggest for the US study. Compared with the published results of the US study, the pooled analysis included fewer cases and controls (34 cases and 90 controls were excluded because 24/48-hour measurements were missing), limited the study period to the year prior to diagnosis rather than the five years immediately prior to diagnosis, restricted the number of residences for which measurements were utilized to one per subject rather than all homes resided in during the five years immediately prior to diagnosis, and used geometric means rather than arithmetic means.

In studies with long magnetic field measurements (24/48-hour), these were chosen as the primary exposure measure. The publication from the Canadian study uses personal measurements, but to achieve consistency with the other studies we chose to use the in-home measurements instead. In the UK, a two-phase measurement strategy was used, according to which 48-hour measurements were conducted when either a shorter measurement (108 minutes) or a characteristic of the residency indicated that EMF exposure was elevated. These measurements were all treated as long measurements because almost all elevated readings would come from 48-hour measurements. None of the adjustments to the measured exposure that were presented in the UKCCS analysis were used in the pooled analysis. (It should be noted that these adjustments had negligible effect.)

*© 2000 Cancer Research Campaign* 

As a summation of all measurements for one subject, over the 24/48 hours, most of the centres used arithmetic means. We decided, however, to use geometric means from all studies, because they are less affected by outliers. For comparison we also analysed the data using arithmetic means. Therefore, each centre provided the geometric means as well as the arithmetic means, regardless of what they used in their original publication.

All centres without long measurements had calculated fields, i.e., calculations of magnetic fields based upon distance between the subject's home and the nearby power line, line characteristics, and load on the line. For these centres calculated fields were evaluated as the primary measure.

We also analysed wire-codes (i.e., a proxy measure of residential magnetic field level, based on the distance and configuration of nearby power lines) for all North American studies. These were classified and analysed according to the original Wertheimer–Leeper scheme (Wertheimer and Leeper, 1982). We also developed a European version of the wire-code, but eventually decided that the differences between the North American and the European distribution systems were too large to make this meaningful. The wire-code analyses, therefore, only included the North American studies.

With respect to the reference time for exposure characterization, there was considerable variation across studies. Residential measurement data were available for various periods from birth to diagnosis. We decided to aim for the average exposure during the last year prior to diagnosis for the cases and the corresponding age for the controls. We achieved this by using the exposure information for the home at the time of diagnosis for the cases and the home lived in by the matched control at the same age; when this information was unavailable we used instead the latest time period prior to diagnosis (Table 1). The reasons were that all studies could provide exposure data specified in this way and that exposure close to date of diagnosis is relevant to the hypothesis that EMF, if anything, would act as a promoter.

All studies utilized a matched case-control design, although the matching variables were not the same in all studies (Table 1). In Finland the original publication reported findings from a cohort study, but in preparation for this pooled analysis a control group was selected and the data were evaluated using a matched case-control design with 3 additional years of follow-up. Because we wanted to use as many as possible of the cases and controls to increase the flexibility of the analysis, we decided to ignore the matching. Instead we included adjustment for age and sex in all analyses, with age classified into one-year groups up to five years of age and then into five year groups. In all analyses, the measurement studies were also adjusted for socio-economic status, according to centre-specific definitions (Table 1). In addition, we adjusted for residence in the eastern or western part of the country in Germany.

One of the aims of this study was to test whether adjustment for any available covariate would have an effect on the summary relative risk estimates. In addition to the covariates included in the basic model, the following factors were available: socioeconomic status, mobility, level of urbanization, detached/not detached dwelling, and level of traffic exhaust. All of these variables were not available in all studies (Table 1). For socioeconomic class, level of urbanization, residential mobility, and traffic exhaust, the basic information and the definitions varied between centres as described in Table 1.

To estimate a summary relative risk across centres, a logistic regression model was applied to the raw data, with centres represented by dummy variables. We did this for measurement studies and calculated field studies separately but also across all studies. In the primary analyses, exposure was categorized in the four levels: < 0.1 μT; 0.1–<0.2 μT; 0.2–<0.4 μT; ≥ 0.4 μT and entered into the model with the use of dummy variables. The wire-code analyses were treated correspondingly. In addition, a similar analysis but with continuous exposure was conducted, the results of which are reported as relative risks per 0.2 μT intervals. This continuous analysis was also the basis for a likelihood ratio test of homogeneity of effects across studies.

### RESULTS

Table 2 gives the absolute numbers of subjects by case/control status, study, and exposure level. In total there are 3247 cases and 10 400 controls. The UK provided by far the largest number of cases, while Denmark had the largest number of controls. In the highest exposure category (≥ 0.4 μT) there were 44 cases and 62 controls, with the largest number of cases from the USA and the largest number of controls from Sweden. Out of the 3247 cases, 2704 (83%) are ALL cases. The US study was restricted to ALL, which explains why the US numbers are the same in the left and right panels of Table 2.

In Table 3 we summarize the primary results for total leukaemia. For each centre the relative risks are estimated by exposure level and with adjustment for the basic potential confounders. Some of the studies are based on small numbers, particularly the highest exposure categories, and in some instances there are zero cases or controls. Although some of the centre-specific relative risk estimates are of little interest in themselves, particularly in the higher categories, all studies still provide information for the summary measures. The last column of the table gives the results of the logistic regression analysis with continuous exposure. The homogeneity test based on the continuous analysis across all nine centres resulted in a $\chi^2$ with eight degrees of freedom of 10.7 corresponding to a *P* value of 0.22. The interpretation is that the variation in point estimates between the studies, is not larger than one would expect from random variability. We compared results for matched versus unmatched analyses to confirm that ignoring the matching did not introduce a bias. Because the results were similar, we only report the unmatched results.

Across the measurement studies, the summary relative risk is estimated at 1.87 (95% CI.: 1.10–3.18) in the highest exposure category, with a corresponding *P* value of 0.01. The two lower categories have estimates close to unity. For the calculated fields studies the summary measure for the top exposure category is 2.13 (0.93–4.88), with a *P* value of 0.04.

In the very last line of Table 3, we give the summary relative risk estimate across all studies, regardless of whether the study is a measurement study or a calculated field study. We consider this an analysis based on the exposure measure that is closest to the specified magnetic field measurement and time period of study defined for the pooled analysis. The relative risk estimates in the two intermediate exposure categories are near the no effect value, while in the top category (≥ 0.4 μT) the relative risk estimate is 2.00 (95% CIs: 1.27–3.13), with a *P* value of 0.002. The continuous analysis gives a relative risk estimate per 0.2 μT of 1.15 (1.04–1.27) with a test for trend *P* value of 0.004.

*© 2000 Cancer Research Campaign*

**Table 2** Absolute numbers of childhood leukaemia cases and controls by study and exposure level

| **Measurement studies** | | | | | | | | | | |
|---|---|---|---|---|---|---|---|---|---|---|
| **Leukaemia cases** | **< 0.1** | **0.1–0.2** | **0.2–0.4** | **≥ 0.4** | **Total** | **ALL cases < 0.1** | **0.1–0.2** | **0.2–0.4** | **≥ 0.4** | **Total** |
| Canada | 174 | 56 | 29 | 13 | 272 | 151 | 50 | 26 | 12 | 239 |
| Germany | 156 | 12 | 5 | 2 | 175 | 130 | 10 | 5 | 2 | 147 |
| New Zealand | 76 | 6 | 4 | 0 | 86 | 64 | 5 | 3 | 0 | 72 |
| UK | 1018 | 38 | 13 | 4 | 1073 | 859 | 34 | 10 | 3 | 906 |
| USA | 418 | 111 | 49 | 17 | 595 | 418 | 111 | 49 | 17 | 595 |
| Total | 1842 | 223 | 100 | 36 | 2201 | 1622 | 210 | 93 | 34 | 1959 |
| Controls | < 0.1 | 0.1–0.2 | 0.2–0.4 | ≥ 0.4 | Total | | | | | |
| Canada | 215 | 53 | 26 | 10 | 304 | | | | | |
| Germany | 380 | 21 | 6 | 2 | 409 | | | | | |
| New Zealand | 72 | 8 | 0 | 0 | 80 | | | | | |
| UK | 2099 | 91 | 26 | 8 | 2224 | | | | | |
| USA | 386 | 95 | 44 | 5 | 530 | | | | | |
| Total | 3152 | 268 | 102 | 25 | 3547 | | | | | |
| Calculated fields studies | | | | | | | | | | |
| Leukaemia cases | < 0.1 | 0.1–0.2 | 0.2–0.4 | ≥ 0.4 | Total | ALL cases < 0.1 | 0.1–0.2 | 0.2–0.4 | ≥ 0.4 | Total |
| Denmark | 830 | 1 | 0 | 2 | 833 | 596 | 0 | 0 | 2 | 598 |
| Finland | 27 | 0 | 1 | 1 | 29 | 25 | 0 | 1 | 1 | 27 |
| Norway | 140 | 6 | 2 | 0 | 148 | 92 | 5 | 2 | 0 | 99 |
| Sweden | 27 | 3 | 1 | 5 | 36 | 17 | 1 | 0 | 3 | 21 |
| Total | 1024 | 10 | 4 | 8 | 1046 | 730 | 6 | 3 | 6 | 745 |
| Controls | < 0.1 | 0.1–0.2 | 0.2–0.4 | ≥ 0.4 | Total | | | | | |
| Denmark | 4736 | 2 | 8 | 0 | 4746 | | | | | |
| Finland | 991 | 19 | 10 | 7 | 1027 | | | | | |
| Norway | 542 | 13 | 7 | 10 | 572 | | | | | |
| Sweden | 438 | 30 | 20 | 20 | 508 | | | | | |
| Total | 6707 | 64 | 45 | 37 | 6853 | | | | | |

**Table 3** Total leukaemia. Relative risks (95% CI) by exposure level and with exposure as continuous variable (RR per 0.2 μT) with adjustment for age, sex, and SES (measurement studies) and East/West in Germany. Reference level: < 0.1 μT. Observed (O) and expected (E) case numbers ≥ 0.4 μT, with expected nos. given by modelling probability of membership of each exposure category based on distribution of controls including covariates.

| **Type of study** | **0.1–< 0.2 μT** | **0.2–<0.4 μT** | **≥ 0.4 μT** | **O** | **E** | **Continuous analysis** |
|---|---|---|---|---|---|---|
| **Measurement studies** | | | | | | |
| Canada | 1.29 (0.84–1.99) | 1.39 (0.78–2.48) | 1.55 (0.65–3.68) | 13 | 10.3 | 1.21 (0.96–1.52) |
| Germany | 1.24 (0.58–2.64) | 1.67 (0.48–5.83) | 2.00 (0.26–15.17) | 2 | 0.9 | 1.31 (0.76–2.26) |
| New Zealand | 0.67 (0.20–2.20) | 4 cases/0 ctrls | 0 cases/0 ctrls | 0 | 0 | 1.36 (0.40–4.61) |
| UK | 0.84 (0.57–1.24) | 0.98 (0.50–1.93) | 1.00 (0.30–3.37) | 4 | 4.4 | 0.93 (0.69–1.25) |
| USA | 1.11 (0.81–1.53) | 1.01 (0.65–1.57) | 3.44 (1.24–9.54) | 17 | 4.7 | 1.30 (1.01–1.67) |
| **Calculated fields studies** | | | | | | |
| Denmark | 2.68 (0.24–30.45) | 0 cases/8 ctrls | 2 cases/0 ctrls | 2 | 0 | 1.50 (0.85–2.65) |
| Finland | 0 cases/19 ctrls | 4.11 (0.48–35.1) | 6.21 (0.68–56.9) | 1 | 0.2 | 1.15 (0.79–1.66) |
| Norway | 1.75 (0.65–4.72) | 1.06 (0.21–5.22) | 0 cases/10 ctrls | 0 | 2.7 | 0.78 (0.50–1.23) |
| Sweden | 1.75 (0.48–6.37) | 0.57 (0.07–4.65) | 3.74 (1.23–11.37) | 5 | 1.5 | 1.31 (0.98–1.73) |
| Summary | | | | | | |
| Measurement studies | 1.05 (0.86–1.28) | 1.15 (0.85–1.54) | 1.87 (1.10–3.18) | 36 | 20.1 | 1.17 (1.02–1.34) |
| Calculated fields studies | 1.58 (0.77–3.25) | 0.79 (0.27–2.28) | 2.13 (0.93–4.88) | 8 | 4.4 | 1.11 (0.94–1.30) |
| All studies | 1.08 (0.89–1.31) | 1.11 (0.84–1.47) | 2.00 (1.27–3.13) | 44 | 24.2 | 1.15 (1.04–1.27) |

In the measurement studies, because several of the relative risk estimates were higher when geometric rather than arithmetic means were employed the data were reanalysed using arithmetic means. Although the summary relative risk for all measurement studies was still elevated 1.59 (1.04–2.45), it was lower than that obtained when the analysis was based on geometric means.

While the primary categorical analyses were based on the predetermined cut off points, we evaluated the robustness of the results by also using other cut off points. With 0.3–<0.4, 0.4–<0.5 and ≥ 0.5 μT as the three highest categories we found, across all studies and for total leukaemia, relative risks of 1.60, 2.54 and 1.75, respectively.

The largest studies and therefore the studies that carry most of the weight in the summations are those from the US, Canada, and the UK. If the US study were to be excluded, the summary estimate for the highest exposure category would be reduced from 2.00 to 1.68 (1.00–2.83; $P = 0.03$). The exclusion of Canada would increase the summary estimates to 2.14 (1.27–3.61), while exclusion of the UK study would increase it to 2.29 (1.41–3.74). Table 3 also gives the expected number of cases in the highest category under the null

© 2000 Cancer Research Campaign

**Table 4** Acute lymphocytic leukaemia. Relative risks (95% CI) by exposure level with adjustment for age, sex, and SES (measurement studies) and East/West in Germany. Reference level: < 0.1 μT.

| **Measurement studies** | **0.1–<0.2 μT** | **0.2–<0.4 μT** | **≥0.4 μT** |
|---|---|---|---|
| Canada | 1.33 (0.85–2.07) | 1.44 (0.79–2.60) | 1.65 (0.68–4.01) |
| Germany | 1.29 (0.58–2.89) | 2.19 (0.62–7.71) | 2.21 (0.29–16.7) |
| New Zealand | 0.71 (0.21–2.44) | 3 cases/0 ctrls | 0 cases/0 ctrls |
| UK | 0.89 (0.59–1.34) | 0.87 (0.42–1.84) | 0.88 (0.23–3.39) |
| USA | 1.11 (0.81–1.53) | 1.01 (0.65–1.57) | 3.44 (1.24–9.54) |
| Calculated fields studies | | | |
| Denmark | 0 cases/2 ctrls | 0 cases/8 ctrls | 2 cases/0 ctrls |
| Finland | 0 cases/19 ctrls | 4.31 (0.50–37.2) | 6.79 (0.74–62.6) |
| Norway | 2.25 (0.78–6.55) | 1.49 (0.30–7.45) | 0 cases/10 ctrls |
| Sweden | 0.88 (0.11–7.19) | 0 cases/20 ctrls | 3.46 (0.84–14.3) |
| Summary | | | |
| Measurement studies | 1.07 (0.87–1.31) | 1.15 (0.84–1.56) | 1.95 (1.14–3.35) |
| Calculated fields studies | 1.42 (0.58–3.45) | 0.84 (0.25–2.81) | 2.23 (0.88–5.65) |
| All studies | 1.08 (0.88–1.32) | 1.12 (0.84–1.51) | 2.08 (1.30–3.33) |

**Table 5** Summary relative risks. (95% CI) for total leukaemia by exposure level based on best available measure with adjustment for potential confounders. Germany also includes East/West adjustment.

| | **0.1–<0.2 μT** | **0.2–<0.4 μT** | **≥ 0.4 μT** |
|---|---|---|---|
| All studies but Finland | | | |
| Age, sex | 1.07 (0.88–1.29) | 1.11 (0.84–1.47) | 1.91 (1.21–2.99) |
| Age, sex, SES | 1.08 (0.89–1.31) | 1.10 (0.82–1.46) | 1.92 (1.22–3.02) |
| All studies but UK | | | |
| Age, sex, SES | 1.18 (0.94–1.48) | 1.15 (0.84–1.58) | 2.28 (1.40–3.71) |
| Age, sex, SES, Urban | 1.13 (0.90–1.42) | 1.09 (0.79–1.50) | 2.24 (1.37–3.67) |
| All studies but UK, Denmark, Finland, and NZ | | | |
| Age, sex, SES | 1.20 (0.96–1.52) | 1.15 (0.83–1.58) | 1.97 (1.19–3.25) |
| Age, sex, SES, type of dwelling | 1.21 (0.96–1.52) | 1.15 (0.83–1.59) | 1.97 (1.19–3.26) |
| All studies but UK and Finland | | | |
| Age, sex, SES | 1.19 (0.95–1.49) | 1.13 (0.83–1.55) | 2.20 (1.34–3.61) |
| Age, sex, SES, mobility | 1.18 (0.94–1.48) | 1.14 (0.83–1.56) | 2.20 (1.34–3.61) |
| Sweden and Germany | | | |
| Age, sex, SES | 1.37 (0.71–2.64) | 1.28 (0.47–3.51) | 3.30 (1.24–8.81) |
| Age, sex, SES, car exhaust | 1.36 (0.70–2.63) | 1.27 (0.46–3.49) | 3.24 (1.22–8.63) |

Reference level: < 0.1 μT.

hypothesis. The total number of excess cases across all studies is 20, the largest number being contributed by the US study.

We then restricted these analyses to ALL. Since the ALL cases make up as much as 83% of all cases and since the controls are the same, the ALL results must be similar to the total leukaemia results. The results in Table 4 show that this is indeed the case, but in the highest exposure category the ALL relative risks are somewhat higher than for total leukaemia.

We also looked separately at other leukaemia to see whether the observed excess risk was restricted to the ALL group. The summary relative risk for other leukaemia was 1.42 in the highest exposure category, but based on only 4 exposed cases.

Next we addressed the issue of a possible effect of adjustment for more covariates. The results of this analysis are given in Table 5. In addition to the centres using different definitions of potential confounders we also faced the problem that all centres did not have data on all potential confounders. When we adjusted for a particular confounder we therefore included only those studies that have data on that confounder. Because of the centre specific differences in relative risks we could not compare the adjusted results calculated from only a subset of the studies to the basic model results calculated from all the studies. Therefore, in Table 5 we present results with and without adjustment for a potential confounder for the group of studies that the estimates are based upon. As can be seen in Table 5, for none of the potential confounders does the adjustment result in anything but minor changes in any of the relative risk estimates.

The final issue is the so-called wire-code paradox. Table 6 has the results according to wire-code categories including a summary estimate for the two North American studies. In the table we also give magnetic field levels for each wire code category. The relative risk for the highest wire-code category is 1.24 (0.82–1.87) so these analyses do not provide evidence for the existence of such a paradox.

### DISCUSSION

We did not find any evidence of an increased risk of childhood leukaemia at residential magnetic field levels < 0.4 μT. We did, however, find a statistically significant relative risk estimate of two for childhood leukaemia in children with residential exposure to EMF ≥ 0.4 μT during the year prior to diagnosis. Less than 1%

*© 2000 Cancer Research Campaign*

**Table 6** Total leukaemia. Relative risks (95% CI) by wire-code with adjustment for age, sex, SES (local definitions) and mobility, number of subjects, and EMF levels based on subset of subjects with measurement on home used in wire code analysis.

| North American studies | UG/VLCC[1] | OLCC[2] | OHCC[3] | VHCC[4] |
|---|---|---|---|---|
| Canada | 1 | 0.98 (0.66–1.46) | 0.75 (0.52–1.10) | 1.59 (0.90–2.82) |
| Case/control | 151/154 | 77/77 | 83/105 | 39/23 |
| USA | 1 | 1.03 (0.73–1.44) | 1.04 (0.71–1.51) | 0.87 (0.47–1.61) |
| Case/control | 177/173 | 119/115 | 88/87 | 24/26 |
| All North American studies | 1 | 1.01 (0.78–1.30) | 0.89 (0.68–1.16) | 1.24 (0.82–1.87) |
| EMF level, median in controls | 0.04 | 0.05 | 0.08 | 0.11 |

[1]Under ground/very low current configuration; [2]Ordinary low current configuration; [3]Ordinary high current configuration; [4]Very high current configuration.

of subjects were in this highest exposure category. The results did not change following adjustment for the potential confounders. In addition, the existence of the so-called wire-code paradox could not be confirmed.

Earlier analyses of the hypothesis of an association between EMF and cancer have sometimes been criticized on the grounds that the findings might be a consequence of so-called data dredging. In order to avoid this and because this work has been a collaborative effort of a rather large group of investigators we specified which primary analyses we planned to do and how to do them before we commenced the analysis; this was before the results of several of the individual studies were known.

The fact that we had access to the raw data from each study gave us two substantial advantages. First, it allowed us to make the data from the various centres as compatible as possible, which was particularly important for the exposure variables. For example, it made it possible to use the same cut-off points in all studies, to use geometric means of the measurements, and to focus on exposure during the year preceding diagnosis. Second, we could arrange data in ways that were of little interest in themselves for some of the individual centres because of small numbers, but still of considerable interest for the total material. In particular this made it possible to analyse, in a consistent way, higher cut-off points than the commonly used 0.2 μT.

For the measurement studies, the findings may have reflected effects of selection bias due to non-participation. Differences were observed in several measures of socioeconomic status between cases and controls, particularly in the US study, with controls generally characterized by higher socioeconomic status than cases. In a recent analysis, Hatch et al found that exclusion of partial or non-cooperative participants from analyses of either in-home magnetic field measurements or wire-codes tended to increase the risk estimates for childhood leukaemia in the US study (Hatch et al, 2000). This was confirmed in the UK study in which there was a moderate association between a deprivation index and measured magnetic fields (UKCCS, 1999). This suggests that at least some of the elevation of risk estimates arose from differential participation of cases and controls.

Exposure measurements from both calculated and measured field studies are subject to error. Time-weighted average in a single 24- or 48-hour period immediately prior to diagnosis may not represent typical levels or the proper metric at the time period that is relevant for assessing risk of leukaemia, if any, and may not reflect the exposure of a child living in the home. Calculated fields are also averages over time and do not take individual characteristics of homes into consideration. Since elevated risk appears to be confined to only the small fraction of children who are highly exposed and since we have no basis for determining the pattern of measurement errors in each study, we cannot reliably infer the underlying risk function that would be consistent with the observed risk pattern.

One feature of our results is the high degree of consistency between the group of studies with measured fields and the group of studies with calculated fields. This may be of significance when considering potential confounders because in the calculated fields studies, the dominant source of exposure is high voltage power lines, while in the measured fields studies internal sources (such as ground currents, household wiring, and exposures from electrical appliances) may predominate. In effect one would not expect the same confounders to be operating in these two types of studies. This may also be of significance when considering selection bias problems, because the calculated fields studies are using population registries in a way that makes selection bias a small issue. In this comparison between the measurement studies and the calculated fields studies, one must keep in mind, however that the calculated fields studies are small and based only on a total of 8 cases with exposure in the highest exposure category.

One of our goals was to see whether controlling for as many putative confounders as possible would change the results, but none of the covariates that we had access to changed the results in any substantial way when included in the models. On the other hand, none of these is an established risk factor for childhood leukaemia. Indeed, knowledge about risk factors for childhood leukaemia is very limited so one cannot exclude the possibility that adjustment for some other variable would have an effect. For the moment we can only conclude that mobility, traffic exhaust, type of dwelling, and urban/rural residency are not important confounders when studying EMF and childhood leukaemia.

An interesting finding in our analysis relates to the so-called wire-code paradox. In an earlier review, an expert committee noted on the basis of the earlier studies that there is a stronger association between markers for EMF exposure and leukaemia risk than between direct measurements and leukaemia risk (National Research Council, 1996). Our data based on subsequent studies do not support this. In fact, the two North American studies show no evidence of increased risk associated with residing in homes in high wire-code categories. It is also worth noting that the measured magnetic fields are low in all the wire-code categories. The reasons for the elevated risk estimates for high wire-code categories in the earlier North American studies are unclear, although considerable potential for bias has been noted for both studies carried out in Denver (Portier and Wolfe, 1998).

The results of numerous animal experiments and laboratory studies examining biological effects of magnetic fields have

© 2000 Cancer Research Campaign produced no evidence to support an aetiologic role of magnetic fields in leukaemogenesis (Portier and Wolfe, 1998). Four lifetime exposure experiments have produced no evidence that magnetic fields, even at exposure levels as high as 2000 µT, are involved in the development of lymphopoietic malignancies. Several rodent experiments designed to detect promotional effects of magnetic fields on the incidence of leukaemia or lymphoma have also been uniformly negative. There are no reproducible laboratory findings demonstrating biological effects of magnetic fields below 100 µT.

Our results have clear implications for future studies. The level of significance that we see for the excess risk at high exposure makes chance an unlikely explanation. Future studies will be of use only if the operation of selection bias and confounding can be adequately addressed, and if there are sufficient numbers with exposure over 0.4 µT.

In summary, for exposure up to 0.4 µT our data demonstrate relative risks near the no-effect level. For the very small proportion (0.8%) of subjects with exposure above 0.4 µT, the data show a two-fold increase, which is unlikely to be due to random variability. The explanation for the elevated risk estimate is unknown, but selection bias may have accounted for some of the increase.

### ACKNOWLEDGEMENTS

We are indebted to the following scientists for input during the planning: Ben Armstrong, Anssi Auvinen, Ray Cartwright, Gerald Draper, Elisabeth Hatch, Kauko Heikkilä, Klea Katsouyanni, Ruth Kleinerman, Markku Koskenvuo, Corrado Magnani, Rolf Meinert, Sam Pattenden, Eero Pukkala, Alberto Salvan, Joachim Schuez, Lorenzo Simonato, and Sholom Wacholder. The study was supported by funding from the Commission of the European Communities, Contract no. BMH4-CT96-0186 and the Swedish Medical Research Council.

### REFERENCES

Dockerty JD, Elwood JM, Skegg DCG and Herbison GP (1998) Electromagnetic field exposures and childhood cancers in New Zealand. *Cancer Causes and Control* **9**: 209–309. Erratum in 1999; **10**: 641.

Feychting M and Ahlbom A (1993) Magnetic fields and cancer in children residing near Swedish high voltage power lines. *American Journal of Epidemiology* **138**: 467–481.

Green LM, Miller AB, Villeneuve PJ, Agnew DA, Greenberg ML, Jiehui L and Donnelly KE (1999) A case-control study of childhood leukemia in Southern Ontario, Canada, and exposure to magnetic fields in residences. *International Journal of Cancer* **82**: 161–170.

Green LM, Miller AB, Agnew DA, Greenberg ML, Jiehui L, Villeneuve PJ and Tibshirani R (1999) Childhood leukemia and personal monitoring of residential exposures to electric and magnetic fields in Ontario, Canada. *Cancer Causes and Control* **10**: 233–243.

Hatch EE, Kleinerman RA, Linet MS, Tarone RE, Kaune WT, Auvinen A, Baris D, Robison LL and Wacholder S (2000) Residential wiring codes and magnetic fields: Do confounding or selection factors distort findings of EMF studies? *Epidemiology* **11**: 189–198.

Linet MS, Hatch EE, Kleinerman RA, Robison LL, Kaune WT, Friedman DR, Severson RK, Haines CM, Hartsock CT, Niwa S, Wacholder S and Tarone RE (1997) Residential exposure to magnetic fields and acute lymphoblastic leukemia in children. *New Engl J Med* **337**: 1–7.

McBride ML, Gallagher RP, Theriault G, Armstrong BG, Tamaro S, Spinelli JJ, Deadman JE, Fincham S, Robson D and Choi W (1999) Power frequency electric and magnetic fields and risk of childhood leukemia in Canada. *American Journal of Epidemiology* **149**: 831–842

Michaelis J, Schuez J, Meinert R, Zemann E, Grigat J-P, Kaatsch P, Kaletsch U, Miesner A, Brinkman K, Kalkner W and Kärner H (1998) Combined risk estimates for two German population-based case-control studies on residential magnetic fields and childhood acute leukemia. *Epidemiology* **9**: 92–94

National Research Council (1996) Possible health effects of exposure to residential electric and magnetic fields. Washington DC: National Academy Press

Olsen JH, Nielsen A and Schulgen G (1993) Residence near high voltage facilities and risk of cancer in children. *British Medical Journal* **307**: 891–895

Portier CJ and Wolfe MS (eds) (1998) National Institute of Environmental Health Sciences Working Goup Report Assessment of health effects from exposure to power-line frequency electric and magnetic fields. Research Triangle Park: NIH publication No. 98–3981

Tynes T and Haldorsen T (1997) Electromagnetic fields and cancer in children residing near Norwegian high-voltage power lines. *American Journal of Epidemiology* **145**: 219–226

UK Childhood Cancer Study Investigators (1999) Exposure to power frequency magnetic fields and the risk of childhood cancer: a case/control study. *Lancet* **354**: 1925–1931

Verkasalo PK, Pukkala E, Hongisto MY, Valjus JE, Järvinen PJ Heikkilä PV and Koskenvuo M (1993) Risk of cancer in Finnish children living close to power lines. *British Medical Journal* **307**: 895–899

Wertheimer N and Leeper E (1970) Electrical wiring configurations and childhood cancer. *Am J Epidemiol* **109**: 273–284

Wertheimer N and Leeper E (1982) Adult cancer related to electrical wires near the home. *International Journal of Epidemiology* **11**: 345–355

*© 2000 Cancer Research Campaign*
